# Long-term efficacy, safety and immunogenicity in patients with rheumatoid arthritis continuing on an etanercept biosimilar (LBEC0101) or switching from reference etanercept to LBEC0101: an open-label extension of a phase III multicentre, randomised, double-blind, parallel-group study

**DOI:** 10.1186/s13075-019-1910-2

**Published:** 2019-05-21

**Authors:** Min-Chan Park, Hiroaki Matsuno, Jinseok Kim, Sung-Hwan Park, Sang-Heon Lee, Yong-Beom Park, Yun Jong Lee, Sang-Il Lee, Won Park, Dong Hyuk Sheen, Jung-Yoon Choe, Chan-Bum Choi, Seung-Jae Hong, Chang-Hee Suh, Shin-Seok Lee, Hoon-Suk Cha, Bin Yoo, Jin-Wuk Hur, Geun-Tae Kim, Wan-Hee Yoo, Han Joo Baek, Kichul Shin, Seung Cheol Shim, Hyung-In Yang, Hyun Ah Kim, Kyung-Su Park, In Ah Choi, Jisoo Lee, Masato Tomomitsu, Seonghye Shin, Jiyoon Lee, Yeong Wook Song

**Affiliations:** 1 Division of Rheumatology, Yonsei University College of Medicine, Seoul, South Korea; 2 Institute of Medical Science, Tokyo Medical University, Tokyo, Japan; 3 Matsuno Clinic for Rheumatic Diseases, Toyama, Japan; 4 Division of Rheumatology, Jeju National University Hospital, Jeju, South Korea; 5 Division of Rheumatology, The Catholic University of Korea, Seoul St. Mary’s Hospital, Seoul, South Korea; 6 Division of Rheumatology, Konkuk University Medical Center, Seoul, South Korea; 7 Division of Rheumatology, Department of Internal Medicine, Yonsei University College of Medicine, Seoul, South Korea; 8 Division of Rheumatology, Department of Internal Medicine, Seoul National University Bundang Hospital, Gyeonggi-do, South Korea; 9 Division of Rheumatology, Gyeongsang National University Hospital, Jinju, South Korea; 10 Division of Rheumatology, Inha University School of Medicine, Incheon, South Korea; 11 Division of Rheumatology, Eulji University School of Medicine, Daejeon, South Korea; 12 Division of Rheumatology, Department of Internal Medicine, Daegu Catholic University School of Medicine, Daegu, South Korea; 13 Division of Rheumatology, Hanyang University Hospital, Seoul, South Korea; 14 Division of Rheumatology, Kyung Hee University Hospital, Seoul, South Korea; 15 Department of Rheumatology, Ajou University Hospital, Suwon, South Korea; 16 Division of Rheumatology, Chonnam National University Medical School and Hospital, Gwangju, South Korea; 17 Department of Medicine, Samsung Medical Center, Seoul, South Korea; 18 Division of Rheumatology, Asan Medical Center, Seoul, South Korea; 19 Department of Internal Medicine, Eulji University College of Medicine, Eulji Hospital, Seoul, South Korea; 20 Division of Rheumatology, Kosin University Gospel Hospital, Busan, South Korea; 21 Division of Rheumatology, Chonbuk National University Hospital, Jeonju, South Korea; 22 Department of Medicine, Division of Rheumatology, Gachon University Gil Medical Center, Incheon, South Korea; 23 Division of Rheumatology, Seoul Metropolitan Government-Seoul National University, Boramae Medical Center, Seoul, South Korea; 24 Division of Rheumatology, Chungnam National University Hospital, Daejeon, South Korea; 25 Division of Rheumatology, Kyung Hee University Hospital at Gangdong, Oriental Hospital, Seoul, South Korea; 26 Division of Rheumatology, Hallym University Sacred Heart Hospital, Kyunggi, South Korea; 27 Division of Rheumatology, The Catholic University of Korea, St. Vincent’s Hospital, Seoul, South Korea; 28 Division of Rheumatology, Chungbuk National University Hospital, Cheongju, South Korea; 29 Division of Rheumatology, Ewha Womans University Mokdong Hospital, Seoul, South Korea; 30 Mochida Pharmaceutical Co., Ltd, Tokyo, Japan; 31 LG Chem Ltd., Seoul, South Korea; 32 Division of Rheumatology, Department of Internal Medicine, Seoul National University Hospital, Jongno-gu, Seoul, 03080 South Korea; 33 Department of Molecular Medicine and Biopharmaceutical Sciences, Graduate School of Convergence Science and Technology and College of Medicine, Medical Research Centre, Seoul National University, Seoul, South Korea

**Keywords:** Etanercept, LBEC0101, Rheumatoid arthritis, Biosimilar, Switch

## Abstract

**Background:**

To evaluate the long-term efficacy, safety and immunogenicity of continuing LBEC0101; the etanercept (ETN) biosimilar; or switching from the ETN reference product (RP) to LBEC0101 in patients with rheumatoid arthritis (RA).

**Methods:**

This multicentre, single-arm, open-label extension study enrolled patients who had completed a 52-week randomised, double-blind, parallel phase III trial of LBEC0101 vs ETN-RP. Patients treated with ETN-RP during the randomised controlled trial switched to LBEC0101; those treated with LBEC0101 continued to receive LBEC0101 in this study. LBEC0101 (50 mg) was administered subcutaneously once per week for 48 weeks with a stable dose of methotrexate. Efficacy, safety and immunogenicity of LBEC0101 were assessed up to week 100.

**Results:**

A total of 148 patients entered this extension study (70 in the maintenance group and 78 in the switch group). The 28-joint disease activity scores (DAS28)-erythrocyte sedimentation rate (ESR) were maintained in both groups from week 52 to week 100 (from 3.068 to 3.103 in the maintenance group vs. from 3.161 to 3.079 in the switch group). ACR response rates at week 100 for the maintenance vs. switch groups were 79.7% vs. 83.3% for ACR20, 65.2% vs. 66.7% for ACR50 and 44.9% vs. 42.3% for ACR70. The incidence of adverse events and the proportion of patients with newly developed antidrug antibodies were similar in the maintenance and switch groups (70.0% and 70.5%, 1.4% and 1.3%, respectively).

**Conclusions:**

Administration of LBEC0101 showed sustained efficacy and acceptable safety in patients with RA after continued therapy or after switching from ETN-RP to LBEC0101.

**Trial registration:**

ClinicalTrials.gov, NCT02715908. Registered 22 March 2016.

**Electronic supplementary material:**

The online version of this article (10.1186/s13075-019-1910-2) contains supplementary material, which is available to authorized users.

## Background

The use of biologic disease-modifying anti-rheumatic drugs (bDMARDs) has contributed markedly to the improvement in the treatment of rheumatoid arthritis (RA) [1]. Indeed, the level of a country’s use of bDMARDs appears to correlate with its control rates of RA [2]. While the reasons for this are complex, access to biologics is important in the ongoing management of RA. However, the cost of some bDMARDs has limited their availability and contributed to restrictive policies around their use [3, 4]. Biosimilars, which are similar but not identical to their innovator bDMARDs, have a cost advantage over innovator products for individuals and healthcare systems, and this may help improve access to therapy [5, 6].

Tumour necrosis factor (TNF)-inhibitors including etanercept (ETN) are effective for the treatment of RA [7], with TNF-α being a well-recognised contributor to the inflammatory changes that occur in RA [8, 9]. ETN was the first approved bDMARD for the treatment of RA [10, 11], and its biosimilar, LBEC0101, was recently approved in Korea (Eucept®) and Japan (Etanercept BS “MA”) in 2018 for the treatment of the same indications as ETN, including RA [12, 13].

Biosimilarity in terms of pharmacokinetics, efficacy and safety should be demonstrated according to the guidelines of regulatory agencies [14, 15]. While establishing the efficacy and safety of long-term use of biosimilars or that of switching from reference product (RP) to biosimilars is not mandatory for regulatory processes, it is very important to examine these parameters in clinical settings for prescribing doctors and patients. Indeed, clinical trials of several biosimilars have shown promising results in terms of long-term efficacy and safety, confirming their potential as an alternative to branded products in patients with RA [16–21].

The pharmacokinetics of LBEC0101 and ETN-RP in healthy male volunteers were similar [22], and the efficacy and safety of LBEC0101 were equivalent to ETN after 52 weeks of treatment in a phase III, randomised, double-blind, parallel-group study [23]. This study was an open-label extension trial of the phase III study [23] and investigated the long-term efficacy, safety and immunogenicity of treatment with LBEC0101 in Korean patients with RA who continued therapy or were switched from ETN-RP to LBEC0101 at the end of the randomised phase of the trial.

## Methods

### Study design

This was a 48-week multicentre, single-arm, open-label extension study conducted at 28 centres in Korea, following a phase III, multicentre, randomised, double-blind, parallel-group study conducted in Korea and Japan [23]. The study received institutional review board approvals, and all procedures were carried out in accordance with the ethical principles of the Declaration of Helsinki and Good Clinical Practice. All patients gave written informed consent prior to inclusion in the study. This study was registered at ClinicalTrials.gov (NCT02715908).

### Patients

Patients who had completed treatment in the preceding randomised double-blind study [23] and required prolonged treatment for RA at the investigators’ discretion were eligible for the extension study. The inclusion criteria for the preceding randomised double-blind study have been previously reported [23].

Patients deemed unable to participate in the extension study because of adverse events (AEs) in the preceding randomised double-blind study or who had ≥ 10 swollen joints (out of the total 66 assessed joints), had ≥ 12 tender joints (out of the total 68 assessed joints), or were pregnant or lactating at the time of the study were excluded. Additional details of exclusion criteria have been reported in the preceding randomised double-blind study [23].

### Drug treatments

In the extension study, all patients self-administered LBEC0101 (50 mg) subcutaneously once per week for an additional 48 weeks, with a 2-week post-treatment follow-up. The needle size of the LBEC0101 pre-filled syringe was changed from 27G in the preceding randomised double-blind study (LG-ECCL002) to 29G in this extension study. The maintenance group included patients who had received LBEC0101 in the preceding randomised double-blind study and continued to receive LBEC0101 in this extension study, and the switch group included patients who had received ETN-RP in the preceding randomised double-blind study. All patients received concomitant methotrexate (MTX) at a stable dose (7.5–15 mg/week). Stable dosages of nonsteroidal anti-inflammatory agents, analgesics and oral/suppository/topical/bronchial/nasal corticosteroids (≤ 10 mg/day prednisone equivalent dose) were also permitted. No DMARDs other than MTX and no intravenous, intramuscular, intra-articular or epidural corticosteroids were allowed.

### Efficacy

The efficacy assessments were mean changes in 28-joint disease activity scores (DAS28)-erythrocyte sedimentation rate (ESR) and DAS28-serum C-reactive protein (CRP) from weeks 0 and 52, as a baseline, to weeks 76 and 100; American College of Rheumatology (ACR)20, ACR50 and ACR70 response rates at weeks 52, 76 and 100 from week 0; remission rate (i.e., DAS28-ESR < 2.6) at weeks 52, 76 and 100; and rate of European League Against Rheumatism (EULAR) response on DAS28-ESR at weeks 52, 76 and 100 compared with week 0.

### Safety

The incidence of AEs and serious AEs (SAEs) were evaluated up to week 102. AEs of special interest that were known as key safety issues for ETN-RP (i.e., infections, sepsis, injection site reactions, malignancies, heart failure, neurological events, tuberculosis, hepatitis B reactivation and interstitial lung disease) were specifically investigated. AEs were coded using MedDRA V.19.0.

### Immunogenicity

Antidrug antibodies (ADAs) and neutralising antibodies at weeks 52, 76 and 100 were analysed by validated electrochemiluminescent immunoassay using Meso Scale Discovery platform (Meso Scale Diagnostics, Rockville, MD, USA). Biotinylated LBEC0101 and SULFO-TAG-labelled LBEC0101 were used to detect ADAs. A neutralising antibody test was performed using biotinylated LBEC0101 and SULFO-TAG-labelled TNF-alpha only when the results were positive for ADAs.

### Statistical analysis

Efficacy analyses were performed on the full analysis set (FAS), defined as all randomised patients who received the investigational product at least once in the extension study and had at least one DAS28-ESR measurement after week 52. All safety analyses were performed on the safety set, which consisted of all randomised patients who received the investigational product at least once in the extension study and completed at least one safety assessment. The primary efficacy endpoint was analysed using analysis of covariance (ANCOVA) with treatment group assigned in the preceding randomised double-blind study and previous use of bDMARDs as fixed factors and week 0 DAS28-ESR score as a covariate. The least square (LS) mean and 95% confidence interval (CI) adjusted by ANCOVA were presented. Missing data were handled using last observation carried forward analysis. Statistical analyses were conducted using SAS version 9.3 (SAS Institute, Cary, NC, USA).

## Results

### Patient disposition and baseline characteristics

**Fig. 1:**
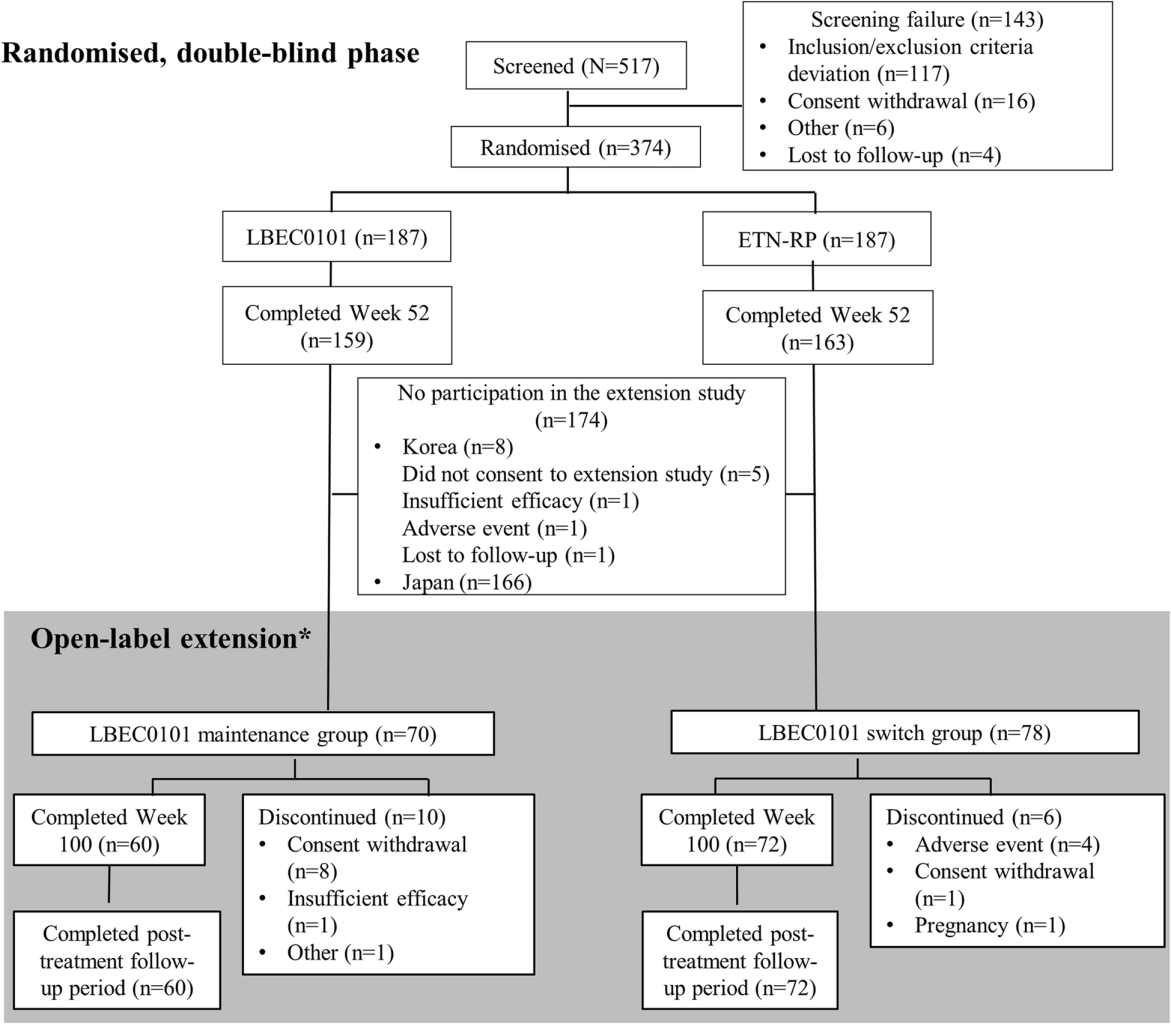
Patient flow chart: Randomisation was carried out in the initial randomised trial only (1:1 ratio to LBEC0101 or ETN-RP), and no further randomisation was carried out in the extension study. *The number of patients enrolled in the extension study is different from the number finishing the phase III study because only Korean patients were enrolled in the extension study. ETN-RP, etanercept reference product

The study flow chart is shown in Fig. 1. Of the 156 Korean patients who completed the preceding randomised double-blind study, 148 participated in the extension study (70 in the maintenance group and 78 in the switch group). One patient failed to complete the post-week 52 DAS28-ESR assessments, leaving 69 patients in the maintenance group and 78 in the switch group included in the FAS.

Characteristics of the FAS at week 0 and week 52 are shown in Table 1. Korean and Japanese patients had a similar disposition in both the LBEC0101 and ETN-RP groups in the preceding, randomised, double-blind study [23]. Although only Korean patients were included in the present study, there were no notable differences in the disposition or RA characteristics between groups at weeks 0 and 52. Both Korean and Japanese patients responded similarly to LBEC0101 and ETN-RP up to week 52; the similarities in RA characteristics were maintained up to week 52 and remained well-balanced between the two groups.

**Table 1:** Baseline patient demographic and disease characteristics at week 0 and week 52 (full-analysis set)

Demographic variable	Maintenance group (*n* = 69)	Switch group (*n* = 78)	Overall (*n* = 147)
Age, years	52.6 ± 11.0	54.5 ± 11.0	53.6 ± 11.0
Female, *n* (%)	52 (75.4)	69 (88.5)	121 (82.3)
Weight, kg	59.9 ± 11.6	57.2 ± 9.6	58.5 ± 10.7
Functional status in RA, *n* (%)			
I	11 (15.9)	15 (19.2)	26 (17.7)
II	50 (72.5)	45 (57.7)	95 (64.6)
III	8 (11.6)	18 (23.1)	26 (17.7)
IV	0	0	0
Time since RA diagnosis, years	8.1 ± 8.4	7.9 ± 7.8	8.0 ± 8.1
Previous use of biologics, *n* (%)			
Week 0^†^	11 (15.9)	8 (10.3)	19 (12.9)
MTX dose, mg/week			
Week 52	12.6 ± 2.7	12.6 ± 2.7	12.6 ± 2.7
Baseline corticosteroids, *n* (%)			
Week 0^†^	57 (82.6)	70 (89.7)	127 (86.4)
Positive rheumatoid factor test result, *n* (%)			
Week 0^†^	48 (69.6)	52 (66.7)	100 (68.0)
Tender joint count from 68 joints			
Week 0^†^	18.4 ± 9.83	18.9 ± 10.68	18.7 ± 10.26
Week 52	1.9 ± 2.48	2.2 ± 2.60	2.0 ± 2.54
Tender joint count from 28 joints			
Week 0^†^	11.5 ± 5.90	11.5 ± 5.76	11.5 ± 5.81
Week 52	1.3 ± 1.83	1.4 ± 1.91	1.3 ± 1.87
Swollen joint count from 66 joints			
Week 0^†^	13.2 ± 8.09	13.5 ± 7.94	13.4 ± 7.98
Week 52	1.0 ± 1.58	1.3 ± 2.04	1.2 ± 1.84
Swollen joint count from 28 joints			
Week 0^†^	9.1 ± 5.42	9.1 ± 5.45	9.1 ± 5.42
Week 52	0.7 ± 1.21	0.9 ± 1.46	0.8 ± 1.34
DAS28-ESR			
Week 0^†^	6.300 ± 0.8949	6.343 ± 0.9170	6.323 ± 0.9039
Week 52	3.068 ± 1.0238	3.161 ± 0.9745	3.117 ± 0.9956
ESR, mm/hour			
Week 0^†^	51.3 ± 21.75	58.4 ± 26.73	55.1 ± 24.69
Week 52	25.4 ± 15.66	29.3 ± 20.88	27.5 ± 18.65
CRP, mg/dL			
Week 0^†^	1.22 ± 1.341	1.52 ± 2.152	1.38 ± 1.818
Week 52	0.20 ± 0.364	0.34 ± 0.770	0.28 ± 0.616
HAQ-DI			
Week 0^†^	1.627 ± 0.7281	1.458 ± 0.7736	1.537 ± 0.7548
Week 52	0.792 ± 0.8241	0.840 ± 0.7972	0.817 ± 0.8075
PtAP			
Week 0^†^	71.67 ± 20.203	65.46 ± 21.722	68.37 ± 21.182
Week 52	21.43 ± 19.788	25.62 ± 22.269	23.66 ± 21.173
PtGADA			
Week 0^†^	69.52 ± 21.619	66.58 ± 20.306	67.96 ± 20.912
Week 52	28.09 ± 23.129	26.71 ± 22.575	27.36 ± 22.769
PhGADA			
Week 0^†^	76.09 ± 14.748	69.12 ± 17.170	72.39 ± 16.400
Week 52	18.16 ± 13.625	16.49 ± 12.324	17.27 ± 12.933

Data are presented as mean ± standard deviation, or number of patients (%)

^†^Week 0 data are given for the population of the extension study only (maintenance group, *n* = 69/switch group, *n* = 78)

*CRP* C-reactive protein, *DAS28-ESR* disease activity score in 28 joints based on erythrocyte sedimentation rate, *ESR* erythrocyte sedimentation rate, *HAQ-DI* Health Assessment Questionnaire Disability Index, *MTX* methotrexate, *PhGADA* physician’s global assessment of disease activity, *PtAP* patient’s assessment of arthritis pain, *PtGADA* patient’s global assessment of disease activity, *RA* rheumatoid arthritis

### Efficacy

**Fig. 2:**
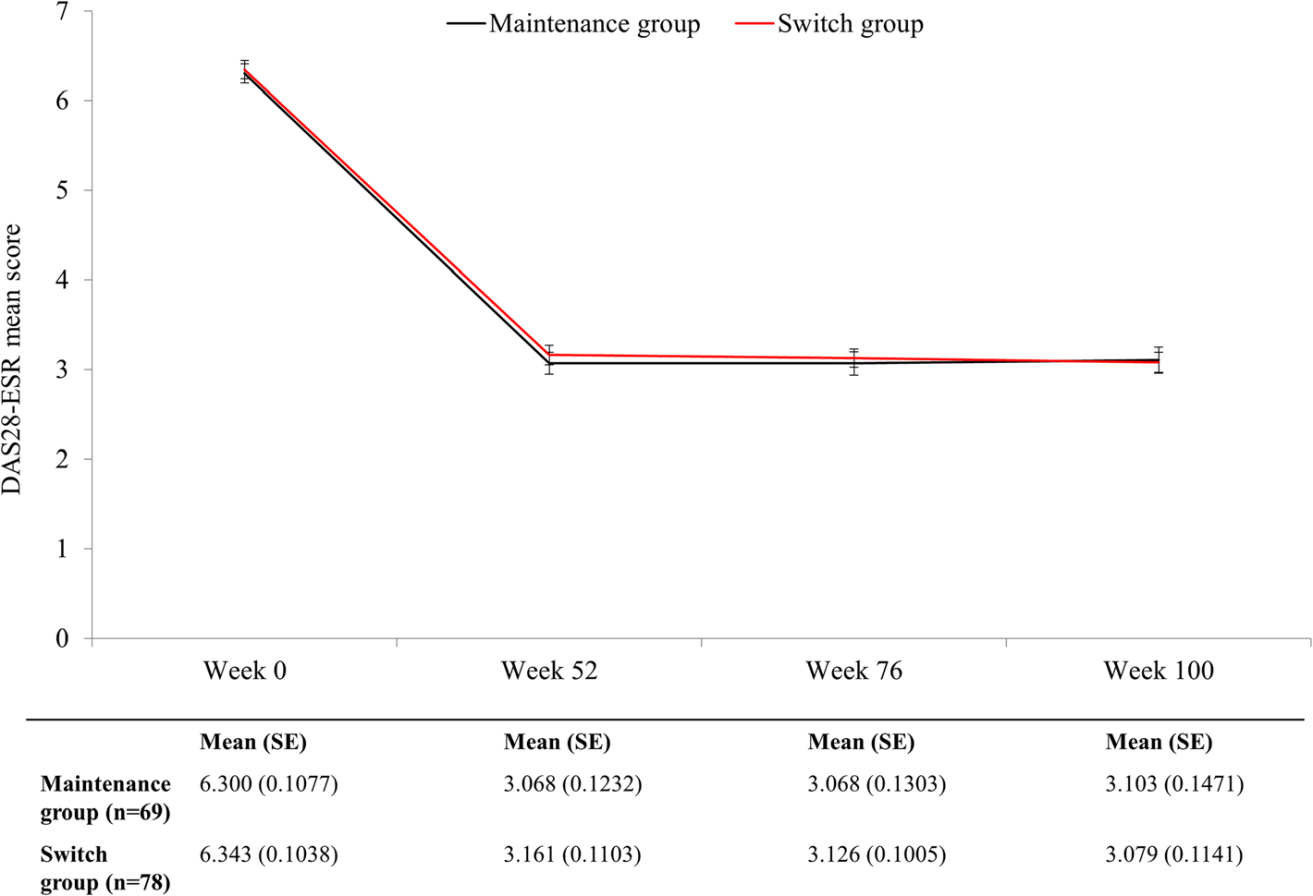
Disease activity score in 28 joints based on erythrocyte sedimentation rate (DAS28-ESR) (full-analysis set). DAS28-ESR mean values at weeks 0, 52, 76 and 100. SE, standard error

During the preceding randomised, double-blind study, efficacy endpoints, including DAS28-ESR and DAS28-CRP scores and ACR response rate, were improved in both the ETN-RP and LBEC0101 groups; the improvements were comparable between the groups at week 52. Improvements in the DAS28-ESR score from week 52 were well maintained throughout this extension phase in both the maintenance and switch groups (Fig. 2): at week 100, the LS mean changes (95% CI) from week 52 were − 0.052 (− 0.314, 0.210) in the maintenance group and − 0.149 (− 0.417, 0.119) in the switch group (estimated treatment difference between groups 0.097 [95% CI − 0.200, 0.393]). At week 100, the corresponding LS mean changes (95% CI) from week 0 were − 3.262 (− 3.567, − 2.957) and − 3.313 (− 3.625, − 3.001) in each group, respectively (estimated treatment difference between groups 0.051 [95% CI − 0.294, 0.395]). The changes in DAS28-CRP score from week 52 to week 100 were also small (LS mean changes 0.240 and 0.138 in the maintenance and switch groups, respectively), indicating that efficacy at week 52 was sustained at week 100.

**Fig. 3:**
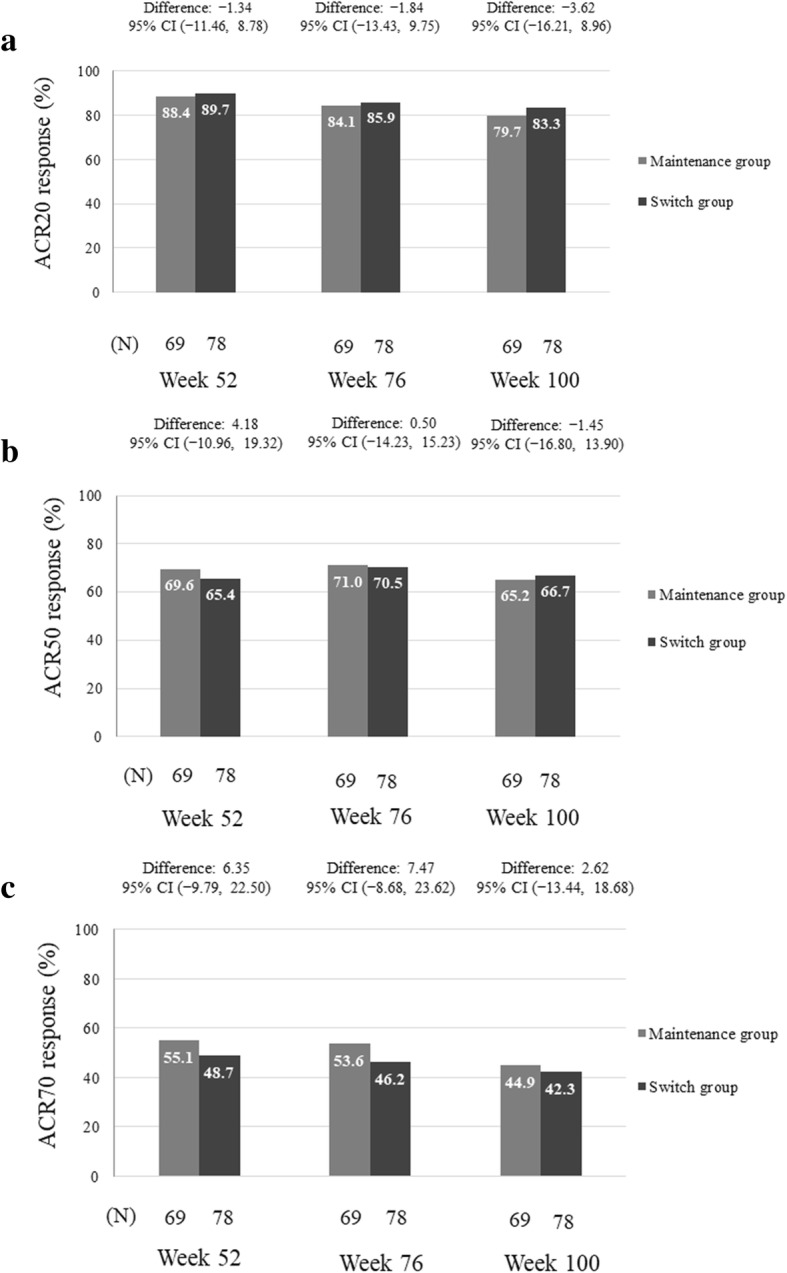
ACR response rates (full-analysis set). **a** ACR20, **b** ACR50 and **c** ACR70 response rates at weeks 52, 76 and 100. ACR, American College of Rheumatology; CI, confidence interval

The ACR20, ACR50 and ACR70 response rates at weeks 52, 76 and 100 are shown in Fig. 3a–c, with no statistically significant differences between the maintenance and switch groups found for any of the results. The improvements in ACR response rates were sustained from week 52 to week 100. The ACR20, ACR50 and ACR70 rates at week 100 based on week 0 were 79.7%, 65.2% and 44.9%, respectively, in the maintenance group, and 83.3%, 66.7% and 42.3% in the switch group.

**Fig. 4:**
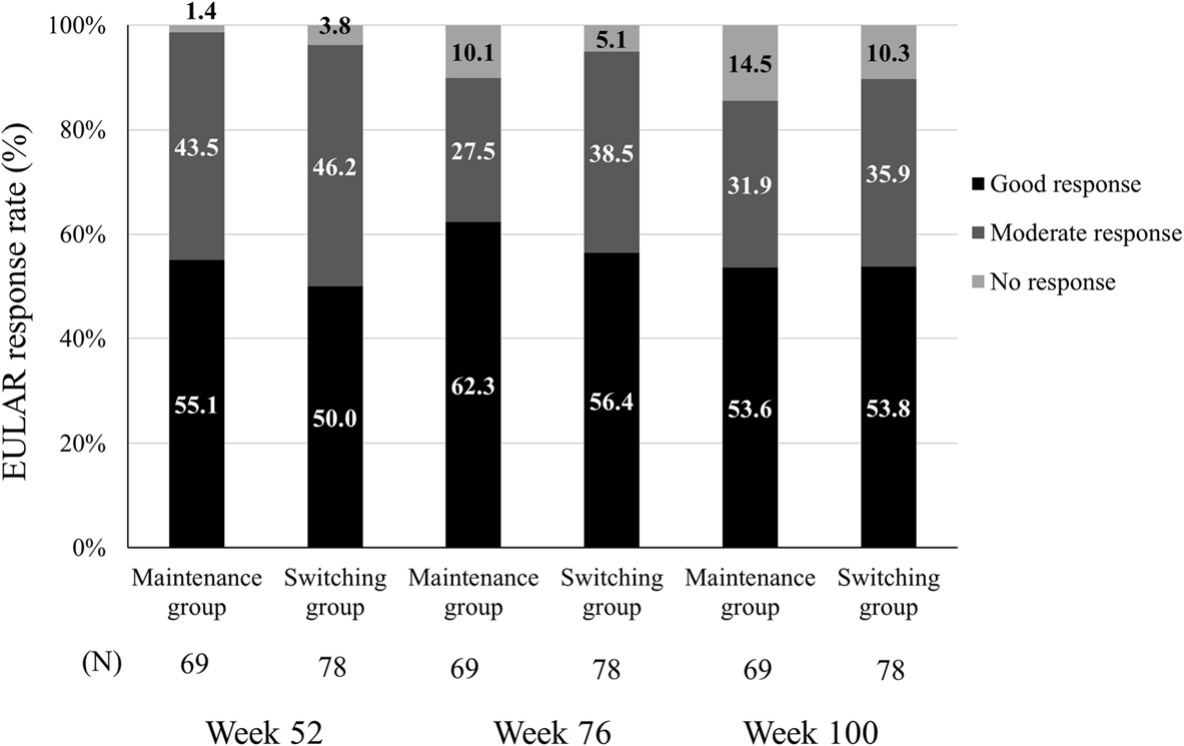
EULAR response rates (full analysis set). EULAR response rates at weeks 52, 76 and 100. EULAR, European League Against Rheumatism

At weeks 52 and 100, the remission rates based on DAS28-ESR < 2.6 in the FAS were 43.5% (30/69) and 36.2% (25/69), respectively, in the maintenance group, while corresponding rates in the switch group were 25.6% (20/78) and 33.3% (26/78). The decrease in the proportion of patients in remission from week 52 to week 100 in the maintenance group was not statistically significant. The rates of EULAR response on DAS28-ESR at weeks 52 and 100 are shown in Fig. 4. The rates of EULAR response and the shift rate of EULAR activity between the two groups were similar.

There were no statically significant differences between the two groups in any of the efficacy results.

### Safety

In the preceding randomised, double-blind study, 90% and 89.7% of patients in the maintenance and switch groups, respectively, reported AEs, and during the extension study, 70.0% and 70.5%, respectively, reported AEs. The overall incidences were 95.7% and 96.2%, respectively, during the entire 100-week study period.

A summary of AEs occurring in the extension study, including AEs experienced by ≥ 5%, is provided in Table 2, with the most common being upper respiratory tract infection, nasopharyngitis and arthralgia. The incidences of SAEs, ADRs, serious ADRs and AEs leading to discontinuation or drug interruption are also shown in Table 2, and all were similar between the two groups. Most AEs during the extension study were mild in severity in both groups, and no deaths occurred. SAEs that occurred in more than one patient during the extension study were acute pyelonephritis (two patients; 1.4%) and arthralgia (two patients; 1.4%). The most frequent AEs and ADRs of special interest were infections and infestations, with no notable differences between the groups. In the preceding randomised double-blind study, injection site reactions were experienced by 23 patients (29.5%), with a total of 164 reactions, in the switch group and by 10 patients (14.3%), with a total of 27 reactions, in the maintenance group. In the present extension study, five patients (6.4%), with a total of 10 reactions, in the switch group and one patient (1.4%), with a total of one reaction, in the maintenance group experienced injection site reactions. Of these, five had experienced similar reactions in the preceding study and one patient in the switch group experienced an injection site reaction for the first time. There were no neurologic events, or cases of heart failure, hepatitis B reactivation, sepsis or interstitial lung disease reported in either group during the extension study.

**Table 2:** Adverse events during the extension study (safety analysis set)

	Maintenance group (*n* = 70)		Switch group (*n* = 78)	
	Number	%	Number	%
All AEs	49	70.0	55	70.5
All ADRs	20	28.6	26	33.3
Serious AEs	6	8.6	8	10.3
Serious ADRs	3	4.3	4	5.1
Any AEs leading to discontinuation	1	1.4	3	3.8
Any AEs leading to temporary drug interruption	13	18.6	8	10.3
AEs experienced by ≥ 5% of patients in either group (by preferred term)				
Upper respiratory tract infection	4	5.7	9	11.5
Nasopharyngitis	7	10.0	4	5.1
Arthralgia	3	4.3	8	10.3
Cough	3	4.3	4	5.1
Active tuberculosis	0	0	0	0
Injection site reaction	1	1.4	5	6.4

*ADR* adverse drug reaction, *AE* adverse event

### Immunogenicity

At the end of the preceding randomised double-blind study (week 52), two (2.9%) and 11 (14.1%) patients in the maintenance and switch groups, respectively, had positive ADA tests, with positive neutralising antibody occurring in one (1.3%) patient in the switch group. During the extension study, one patient in the maintenance group (1.4%) and one patient in the switch group (1.3%) had new positive ADA test results. No patients had new positive neutralising antibody during the extension study. The immunogenicity results for the different study periods are shown in Additional file 1: Table S1.

## Discussion

This open-label extension study evaluated the long-term efficacy, safety and immunogenicity of LBEC0101 in Korean patients with RA who were previously treated with ETN-RP or LBEC0101 for 52 weeks during a phase III, randomised, double-blind study which included both Korean and Japanese patients. We compared clinical parameters for up to 100 weeks in patients who either continued LBEC0101 or switched from ETN-RP to LBEC0101 after completion of the preceding study. Equivalent efficacy and comparable safety profiles for LBEC0101 and ETN-RP were demonstrated in the 52-week study [23]. The improvements in DAS28-ESR, DAS28-CRP and ACR response rate shown during the preceding study were sustained in both the maintenance and switch groups, and similar proportions of patients in both groups achieved a good or moderate EULAR response and remission. Overall, these results confirm that the efficacy previously shown for LBEC0101 extended to week 100 and that efficacy was maintained for patients who switched to LBEC0101 from ETN-RP.

The safety profile was maintained with no notable differences between groups and no new safety concerns. Regarding AEs of special interest, no neurologic events, cases of heart failure, hepatitis B reactivation, sepsis, or interstitial lung disease were reported in either group. Latent TB was confirmed but there were no cases of active TB and no notable between-group differences in AEs of known key ETN-RP safety issues.

The incidence of injection site reactions was lower than that in the preceding study, which may be attributable to the reduced injection needle size used in the extension study [24, 25]. Most injection site reactions in the ETN-RP group of the preceding study occurred in the early stage of the treatment period. However, in the extension study, the incidence in the switch group was low in the early stage after the switch, suggesting that ETN-RP and LBEC0101 are similar in this regard. One important factor to consider is whether switching increases the risk of ADA, which can lead to immunological reactions and decreased drug efficacy [26]. One patient in each group had new positive ADA test results and the incidence of ADA rarely increased after week 52 (Additional file 1: Table S1), demonstrating that no new immunogenicity concerns arose after the switch.

Several studies have documented continued efficacy and safety after switching from RPs to biosimilars, and after long-term treatment [18–21]. In a study by Emery et al. [18], patients received the ETN biosimilar SB4 for 48 weeks in an open-label extension after an initial 52-week randomised controlled trial of SB4 or ETN. Efficacy and safety were maintained until week 100 in both the maintenance and switch groups. The PLANETRA extension study reported that switching from the infliximab RP to CT-P13 did not decrease efficacy or change the safety profile [20]. In a study comparing adalimumab and its biosimilar SB5, efficacy and safety were maintained after switching, albeit after a shorter overall duration of treatment (52 weeks vs 100 weeks in our study) [19].

A lower than expected retention rate has been observed in RA patients switched from the infliximab RP to CT-P13 in recent open-label [27] and real-life [28] switch studies and is thought to be attributable to the nocebo effect. The results of the present extension study provide no evidence for the nocebo effect, as the discontinuation rate in the switch group was not higher than that in the maintenance group (7.7% and 14.3%, respectively).

Key limitations were that the assessment was only conducted up to week 100 and that only the Korean patients were included. Therefore, efficacy and safety for longer-term usage or in other ethnic populations should be evaluated in post-marketing surveillance studies. It should also be noted that the data from our study were analysed and presented as grouped patients; variations in efficacy may occur, meaning that the results of this study may not apply to each individual patient.

Both the efficacy and safety of switching to LBEC0101 need to be confirmed during post-marketing surveillance. Our study only included patients who continued LBEC0101 or switched from ETN-RP to LBEC0101. Biosimilars are only considered interchangeable when it is shown that the risk of diminished safety and efficacy when switching is not greater than when the RP is used alone. Additionally, US FDA guidelines state that switching studies should evaluate switching between interchangeable medications two or more times [26, 29]. Therefore, further studies that include patients who have undergone two or more switching intervals are required. Recent consensus-based recommendations for biosimilar treatment of rheumatological diseases recommend continued monitoring of their safety and efficacy by assessing data from multiple biosimilar/RP switches [30].

## Conclusions

In conclusion, long-term administration of LBEC0101 was associated with ongoing efficacy in patients with RA. Patients who switched from ETN-RP at the end of the preceding randomised double-blind study showed persistent efficacy of therapy after switching to LBEC0101. LBEC0101 was well tolerated in both the maintenance and switch groups, with no new safety concerns identified.

## Additional file

Additional file 1: **Table S1.** Immunogenicity data. (PDF 55 kb)

## Abbreviations

ACR: American College of Rheumatology
ADA: Antidrug antibody
AE: Adverse events
ANCOVA: Analysis of covariance
bDMARD: Biologic disease-modifying anti-rheumatic drug
CI: Confidence interval
CRP: C-reactive protein
DAS28: 28-joint disease activity
ESR: Erythrocyte sedimentation rate
ETN: Etanercept
EULAR: European League Against Rheumatism
FAS: Full analysis set
LS: Least square
MTX: Methotrexate
RA: Rheumatoid arthritis
RP: Reference product
SAE: Serious adverse event
TNF: Tumour necrosis factor
