# Supplementary material for: Long-term efficacy, safety and immunogenicity in patients with rheumatoid arthritis continuing on an etanercept biosimilar (LBEC0101) or switching from reference etanercept to LBEC0101: an open-label extension of a phase III multicentre, randomised, double-blind, parallel-group study
Source: Arthritis Res Ther. 2019 May 21;21:122. doi: 10.1186/s13075-019-1910-2 (PMC6528252; doi:10.1186/s13075-019-1910-2)
Supplement: Supplementary file 1 — Table S1. Immunogenicity data. (PDF 55 kb) [file 13075_2019_1910_MOESM1_ESM.pdf]

Table S1. Immunogenicity data

Study period	Parameter	Maintenance group (n=69)	Switch group (n=78)	Overall (n=147)
Week 0–52	Any of new positive cases of immunogenicity	2 (2.9)	11 (14.1)	13 (8.8)
	Anti-drug antibody	2 (2.9)	11 (14.1)	13 (8.8)
	Neutralising antibody	0	1 (1.3)	1 (0.7)
Week 52–100	Any of new positive cases of immunogenicity	1 (1.4)	1 (1.3)	2 (1.4)
	Anti-drug antibody	1 (1.4)	1 (1.3)	2 (1.4)
	Neutralising antibody	0	0	0
